# Measuring inequality: tools and an illustration

**DOI:** 10.1186/1475-9276-5-5

**Published:** 2006-05-22

**Authors:** Ruth FG Williams, DP Doessel

**Affiliations:** 1 School of Applied Economics, and Centre for Strategic Economic Studies, Victoria University, Melbourne, Australia; 2 Queensland Centre for Mental Health Research, The Park – Centre for Mental Health, Qld; and School of Population Health, The University of Queensland, Australia

## Abstract

**Background:**

This paper examines an aspect of the problem of measuring inequality in health services. The measures that are commonly applied can be misleading because such measures obscure the difficulty in obtaining a complete ranking of distributions. The nature of the social welfare function underlying these measures is important. The overall object is to demonstrate that varying implications for the welfare of society result from inequality measures.

**Method:**

Various tools for measuring a distribution are applied to some illustrative data on four distributions about mental health services. Although these data refer to this one aspect of health, the exercise is of broader relevance than mental health. The summary measures of dispersion conventionally used in empirical work are applied to the data here, such as the standard deviation, the coefficient of variation, the relative mean deviation and the Gini coefficient. Other, less commonly used measures also are applied, such as Theil's Index of Entropy, Atkinson's Measure (using two differing assumptions about the inequality aversion parameter). Lorenz curves are also drawn for these distributions.

**Results:**

Distributions are shown to have differing rankings (in terms of which is more equal than another), depending on which measure is applied.

**Conclusion:**

The scope and content of the literature from the past decade about health inequalities and inequities suggest that the economic literature from the past 100 years about inequality and inequity may have been overlooked, generally speaking, in the health inequalities and inequity literature. An understanding of economic theory and economic method, partly introduced in this article, is helpful in analysing health inequality and inequity.

## Background

"It is not the business of the botanist to eradicate the weeds.

Enough for him to tell us how fast they grow",

C. Northcote Parkinson,

cited by Cowell (1995), p. *ix*

"Distribution", "equality" and "equity" are often used as if their meanings are self-evident, an observation which is false. At times, these concepts are used interchangeably, particularly in some parts of the literatures concerned with health services [1]. Such usage is not helpful. There are important distinctions of meaning between these terms. Occasionally, "equality" and "equity" are also applied interchangeably when qualifying some other concept, such as "access". This is another unhelpful lack of distinction.

Another area where clarity is lacking is in inequality measurement. Health inequality and inequity have recently been discussed and examined extensively, [21,30] for example, and comprehensively reviewed [22]. However, contrary conceptual and empirical evidence about the causes of health inequality also exists [26-28]. The difference in evidence involves differing approaches to measuring and analysing inequality. The present article considers some aspects of how an understanding of economic method assists in analysing health inequality and inequity.

### Distribution, equality and equity

Economic studies of personal distributional issues commonly examine the distribution of income or of aggregate consumption expenditure. However, an economic approach can also be applied to a sub-set of consumption expenditure, such as that on medical services or other health sector services. Conventional economic measures of distribution of income are applicable to any sub-set of income or consumption. This is, of course, possible because of the conceptual macroeconomic relationships underlying National Income Accounting (the economic basis for determining National Income and the Gross Domestic Product), which demonstrates that expenditures, and sub-groups thereof, are the dual, or the identity, of income.

By implication, national accounting relationships enable the application of the numerous income distribution measures to the relevant sub-set of National Income under study here, viz. health, and also to health expenditures. These concepts are applied here to a distribution on psychiatric services, a sub-group of medical services. Of course, the approach here is applicable to any medical sub-group, and to non-medical groups [2].

Inequality in psychiatric service utilisation *per se *is a relevant quantity of interest because of what it reveals indirectly about the relationship between inequality of health service utilisation and psychiatric need. In examining the nature of mental health inequalities, a limitation is that unit record data on psychiatric service utilisation usually are not available (or if such data are available, they are not available in sufficient quantity for statistically powerful analysis). Thus one cannot determine, at an individual level, how well utilisation relates to need. Analysis of aggregated (or grouped) data is therefore necessary. At this level, it is very pertinent that several epidemiological studies about mental health at the regional level of analysis [23,25,29] reveal that mental health status, i.e. need, is relatively invariant at a regional level. The implication is that, if regional differences are detected in the equality of psychiatric service utilisation, then one must suspect that the difference is arising for reasons other than psychiatric need. Two economic constraints on service utilisation or consumption, *viz. *price (including time price and the costs of distance) and income, are possible "suspects". Measuring inequality in psychiatric service utilisation is, therefore, a relevant quantity of interest.

Consider a hypothetical distribution of any quantifiable variable (i.e. the variable may be personal income or personal consumption but it may also be health expenditures or quantities of psychiatric services). The term, distribution, refers to the frequency with which the data are distributed in the population or sample under study. In other words, we are concerned with analysing how a total (of psychiatric services, say) is distributed amongst the various people who comprise the community. At a general level, we are concerned with the following:

PS = ps_1 _+ ps_2 _+ ..., ps_*n*_, or      (1)

where PS is the total of all psychiatric services produced and consumed; and ps_*i *_is the quantity of psychiatric services consumed by person *i*; and *i *= 1, 2, ..., *n*.

It is clear that such a total of psychiatric services (and the distribution thereof) can be analysed in various time periods. Thus, equation (1) holds for a particular time period t, where t = 1, 2, ..., t. A subscript on PS below indicates various time periods.

PS_t = 1 _= (1, 0, 2, 0, 3, 0, 4, 0, 5, 0, 6)     (2)

The subscript 1 associated with PS refers to a particular time period 1. Equation (2) presents the distribution of 21 psychiatric services in total.

Now consider this same eleven-person community at a later time, period 2. In this subsequent time period, the distribution of consumption is as follows:

PS_t = 2 _= (2, 0, 4, 0, 6, 0, 8, 0, 10, 0, 12)     (3)

Distribution (3) is different from the previous distribution in that each person who has a non-zero consumption of psychiatric services has twice as many services as in Distribution (2). Thus, the total number of psychiatric services produced in this time period is 42, which is, in absolute terms, twice that of the 21 services in the previous time period 1. However, the **relative **levels of consumption remain the same as that in period 1.

Examine now another distribution of psychiatric services in this eleven-person community at time period 3:

PS_t = 3 _= (3, 4, 4, 4, 4, 4, 4, 4, 4, 4, 3)     (4)

This distribution of psychiatric services involves more equal consumption of these services, although the total number of services, 42, is the same as the total in time period 2. This distribution of psychiatric services involves more equal consumption of these services, although the total number of services, 42, is the same as the total in time period 2. Note that in Distribution (4), individuals who previously consumed zero psychiatric services are now consuming some psychiatric services.

A fourth distribution of psychiatric services exists in time period 4, but there is now a 12-person society, and that additional person consumes three psychiatric services. Thus, there are 46 services distributed within the larger 12-person community. Apart from these two variables, the consumption of psychiatric services is the same, for the first 11 people, as it was in period 2. Thus, the distribution of the 46 psychiatric services in period 4 is as follows:

PS_t = 4 _= (2, 0, 4, 0, 6, 0, 8, 0, 10, 0, 12, 3)     (5)

Thus we have described four distributions of psychiatric services. A question that follows from the above is this: are these distributions the same, or are they different? This question is too general and can lead to an ambiguous conclusion.

Let us now compare the distribution PS_t = 1 _with the distribution PS_t = 2_. These two distributions are different in terms of absolute consumption, in that each person consuming some psychiatric services is consuming twice as many services compared with their consumption in period 1. The total number of psychiatric services in period 2 is also twice as high as it was in period 1. However, the relative consumption in the two periods is the same. So, in an absolute sense the distributions are different, but in a relative sense, they are the same.

Having presented these distributions, let us consider some economic interpretations. Economic meaning can be attached to the statistical measurement of equality. It (equality) is a concept that can be measured and described with the tools of positive economics, and its presence can be verified or falsified. For example, the share of total psychiatric services of each individual in a population can be calculated. If each individual's share of the total is not the same as each other's share, then the distribution is not equal.

A distribution may be characterised by equality, or by degrees of equality; it may also be characterised by degrees of equity. The equity of a distribution is difficult to judge, even when individuals' shares (say, in total psychiatric services) are equal. This is usually because other characteristics, e.g. illness, may be relevant. The equitability of a distribution is also difficult to judge when individuals' shares are unequal. There is no evidence provided here about whether Distribution (4) above is more equitable than Distribution (3). Lorenz [3] discussed this point. Depending on various value judgements, additional information about each person's mental health status and income level, or other factors deemed relevant, is required to judge which of distribution (2), (3), (4) or (5) is "fairer".

In some accounts of these points [4,5], there are implicit value judgements in addition to the explicit statements commencing the study. This tendency, which occurs in the majority of studies in the health services literatures, makes it difficult to know the grounds upon which welfare judgements about the achievement of "more" or "less" equality / equity are being made.

The conventional statistical measures of location and dispersion and the economic measures of inequality will now be discussed.

## Method

Describing a distribution statistically requires determining the location and dispersion of the data. The location of a data set refers to the absolute levels of the data. Dispersion refers to the extent of inequality between observations, relatively speaking. The distinction between these two concepts can be understood particularly clearly when comparisons of data sets are made. For example, in the distributions PS_1 _and PS_2 _above, the absolute level of the numbers of psychiatric services in those distributions differs (i.e. their location differs), but the relative distribution of psychiatric services of each distribution (i.e. the dispersion) is equivalent. The distinction between the concept of the location of a distribution (and its measurement), and the concept of dispersion (and its measurement) is long-standing in statistical discourse [e.g. [6]] and in economics discourse [e.g. [7]].

### Measures of location and dispersion

Let us consider the measurement of the concept of location. Location is a dimension (measure or index) of central tendency, i.e. of where the distribution is positioned. The arithmetic mean is the most commonly encountered of such measures; other means that are less frequently encountered are the geometric mean and the harmonic mean. The median and its various derivative measures, e.g. the percentile, quartile and quintile, as well as the mode, are other measures of central tendency. All such measures are concerned with determining where in numeric space a distribution is located.

Dispersion (or the "relative distribution", described loosely) is the other dimension (or descriptor) of a distribution. Measures of dispersion are concerned with the variability or inequality in the distribution. Such measures include the following: the range (and other measures derived from the range, such as the interdecile and the interquartile); and also various measures of deviation from, say, the mean e.g. the variance, the mean deviation and the standard deviation; and other deviation measures, such as the mean difference, and the relative mean deviation. Other measures of dispersion that involve a standardised unit of measurement include the coefficient of variation (first defined by Pearson), the Lorenz curve, and the Gini coefficient of concentration [3,6-8] The measures are discussed again below.

All such measures may be referred to as "univariate" measures of dispersion. "Bivariate" measures of a distribution [10-12] refer to measures of dispersion of a bivariate relationship, i.e. of the variable under study and also an additional variable. In other words, a second dimension that is associated with the distribution, such as a demographic or socio-economic dimension, is included. In most cases, ranked data are involved. An example of such a measure is the relative index of inequality [9,10]. A very common bivariate measure in the health literature is the concentration index [13,14].

There is another important class of measures, which involve an explicit theoretical basis for the economics of inequality. Early last century, equality measures that are based upon a social welfare function were proposed [15,16]. Such measures are relevant to economic studies of inequality because statistical measures of dispersion do not necessarily measure the economic concept of inequality. Atkinson's index, or measure, links a social welfare function to a concept of equality. Atkinson employs the notion of measuring the cumulative deviation from the "equally distributed equivalent income", which is the "level of income per head which if equally distributed would give the same level of social welfare as the present distribution" [16]. (In all discussions here, the word "psychiatric services" can replace "income".) In Atkinson's measure, a parameter for assumed levels of inequality aversion ε is used. As the value of ε rises, relatively more weight is attached to inequality at the lower end of the distribution, and relatively less at the upper end. When ε is very large, inequality is sensitive only to transfers among the lowest levels; when ε is zero, transfers (of, say, income or health services) have zero weight, and distributions are ranked only in terms of the total level of income (or total level of health/psychiatric services). Cowell [7] shows the relationship between the distribution of income and the distribution of utility via a four-quadrant diagram, and demonstrates how differing inequality measures can be derived from various values of ε.

Another measure in this class involves a different conceptual basis based upon the entropy concepts used in information theory. Theil's entropy index [17] is specifically useful for its decomposition properties [7].

Mathematical functions exist for all such measures, and the properties of those formulae can be evaluated against criteria about what properties are desirable for an inequality measure [e.g. [7]]. Note that while specialised equality indexes are usually regarded as superior to simple statistical measures, suitable data are necessary.

This discussion of more specialised measures has introduced the idea that equality measures all have a welfare basis and that even descriptive (summary) statistics, for which there may be no explicit welfare basis, have an implicit welfare basis. Welfare weights are built into their formulae, weightings that are arbitrary in terms of their economic meaning, and the welfare implications are discussed elsewhere [7,18,19,24].

## Results

Some of the measures of location and dispersion just discussed are demonstrated in this Section using the data for the four distributions that were provided in the previous Section, viz. PS_t = 1_, PS_t = 2_, PS_t = 3_and PS_t = 4_.

Table 1 presents measures of the absolute level of consumption of psychiatric services, the measures of the relative levels of consumption, or dispersion, for each of these distributions. Note that two measures of the location of these distributions are given in Columns (2) and (3) of the Table, the mean and the median. The measures of dispersion are provided in the next columns of the Table: the range; the standard deviation; the coefficient of variation; the relative mean deviation; the Gini coefficient; Theil's entropy index; and the Atkinson measure, with two assumed values for the inequality aversion parameter, ε = 0.25; and ε = 0.75. See Columns (4) to (10).

**Table 1:** Some measures of dispersion of four illustrative distributions of psychiatric services

**Absolute and Relative Consumption of Psychiatric Services in Each Time Period**		Total Psych. Services	**Measures of Location**		Measures of Dispersion							
		**(1)**	**MN** **^(a)^** **(2)**	**MD** **^(a)^** **(3)**	RNG ^(a)^ (4)	SD ^(a)^ (5)	CoV ^(a)^ (6)	RMD ^(a)^ (7)	Gini ^(a)^ (8)	Theil ^(a)^ (9)	Atkinson ^(a)^ (10)	
											A_0.25_	A_0.75_
**PS_1_**	**TIME PERIOD 1 (11 persons)** ***Absolute Consumption of Psych. Services*:** **1, 0, 2, 0, 3, 0, 4, 0, 5, 0, 6**	21 units	**1.91**	**1.00**	0–6	2.256	1.18	-	-	-	-	-
	***Relative Consumption of Psych. Services (%): *4.8, 0, 9.5, 0, 14.3, 0, 19.0, 0, 23.8, 0, 28.6**	100%	**-**	**-**	-	-	-	-0.005	0.64	0.319	0.2106	0.8538

**PS_2_**	**TIME PERIOD 2 (11 persons)** ***Absolute Consumption of Psych. Services*:** **2, 0, 4, 0, 6, 0, 8, 0, 10, 0, 12**	42 units	**1.91**	**2.00**	0 – 12	4.513	1.18	-	-	-	-	-
	***Relative Consumption of Psych. Services (%): *4.8, 0, 9.5, 0, 14.3, 0, 19.0, 0, 23.8, 0, 28.6**	100%	**-**	**-**	-	-	-	-0.005	0.64	0.319	0.2106	0.8550

**PS_3_**	**TIME PERIOD 3 (11 persons)** ***Absolute Consumption of Psych. Services*:** **3, 4, 4, 4, 4, 4, 4, 4, 4, 4, 3**	42 units	**3.82**	**4.00**	3 – 4	0.405	0.11	-	-	-	-	-
	***Relative Consumption of Psych. Services (%): *7.1, 9.5, 9.5, 9.5, 9.5, 9.5, 9.5, 9.5, 9.5, 9.5, 7.1**	100%	**-**	**-**	-	-	-	-0.005	0.03	0.002	0.0019	0.0047

**PS_4_**	**TIME PERIOD 4 (12 persons)** ***Absolute Consumption of Psych. Services*:** **2, 0, 4, 0, 6, 0, 8, 0, 10, 0, 12, 3**	45 units	**3.75**	**2.50**	0 – 12	4.309	1.15	-	-	-	-	-
	***Relative Consumption of Psych. Services (%): *4.4, 0, 8.9, 0, 13.3, 0, 17.8, 0, 22.2, 0, 26.7, 6.7**	100%	**-**	**-**	-	-	-	0.000	0.38	0.286	0.2109	0.9942

Notes: (a) MN – mean; MD – median; RNG – range; SD – standard deviation; CoV – coefficient of variation; RMD – relative mean deviation; Gini – Gini coefficient; Theil – Theil index of entropy; Atkinson – Atkinson measure of inequality.

It is crucially important to keep in mind that the Atkinson measure is included here by way of illustration, but actually it is inappropriate to apply this index to distributions where zeros are involved. Where non-zero data sets are available, the Atkinson measure is a powerful index, as both an inequality measure and as an index of the potential welfare gains from redistribution of the variable under study.

The two values of ε represent a somewhat high value (relevant where aversion to inequality is relatively high) and a somewhat low value of ε (relevant where aversion to inequality is relatively low). In regard to various possible values of ε, the "leaky bucket" mental experiment, devised by Atkinson, illustrates the meaning of ε [18,20]. Measured inequality is greater the higher is the value of ε and the magnitude of a welfare gain is greater the larger the value of ε, and also the more unequal is the distribution at the *status quo*.

**Figure 1:**
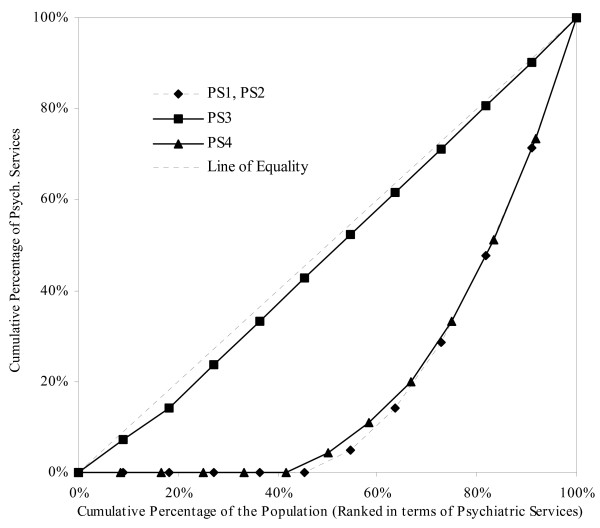
Lorenz Curves for illustrative data on four distributions of psychiatric services

Figure 1 shows four Lorenz curves for the four distributions of psychiatric services, PS_1_, PS_2_, PS_3 _and PS_4_, where the cumulative percentage of total psychiatric services with the cumulative percentage of the population, ranked from "lowest" to "highest". The diagonal depicts a perfectly equal distribution of psychiatric services. The line of perfect inequality corresponds with the (right-hand) y-axis and the x-axis, i.e. it is _+_-shaped. Thus, the degree of inequality of the four distributions lies between perfect equality and perfect inequality.

When the distributions in Table 1 and Figure 1 are examined in order to measure the degree of equality in these distributions, one observes that PS_t = 3 _is the most equal distribution on the basis of all the measures, except the relative mean deviation. Note that the Gini coefficient, the entropy index and both Atkinson measures (A_0.25 _and A_0.75_) for PS_t = 3 _are almost zero. Note also that the Lorenz curve for PS_t = 3 _is closest to the Line of Equality and is wholly above the other three Lorenz curves. The Lorenz curves for PS_t = 1 _and PS_t = 2 _coincide, as expected. However, the Lorenz curves for PS_t = 1 _and PS_t = 2 _merge or intersect with the Lorenz curve for PS_4_. This suggests that PS_t = 4 _is more equal at lower levels of consumption of psychiatric services but that the extent of inequality occurring at higher levels of consumption for PS_t = 1_, PS_t = 2 _and PS_t = 4 _is approximately equivalent.

Note also that a disparity exists between the Gini coefficient and the other measures: the Gini coefficients for PS_t = 1_, PS_t = 2 _and PS_t = 4_indicate relatively large differences in the degree of inequality in those distributions, while similar degrees of inequality are not reflected in either Theil's entropy index or the Atkinson measures.

All the measures of inequality in Table 1 and Figure 1 (except the relative mean deviation) confirm the visual observation of the data: that the relative distribution of PS_3 _is a nearly equal distribution, and is more equal than the other three distributions. (If these data were samples, then no statistical significance could be attached to such a conclusion, as standard errors and confidence intervals are not available. However, these data are complete enumerations, of populations of 11 persons, and 12 persons.) However, the relative mean deviation "tells" a different equality "story": PS_t = 1_, PS_t = 2 _and PS_t = 3 _are equal in terms of relative distribution.

## Discussion

The discussions of "equality" versus "equity" here do not enter into the deep debates and dialogues about which inequalities are unfair and should be considered inequities. Our purpose is somewhat simpler: to seek to infuse those discussions with empirical content, by encouraging the measurement of inequality. A comprehensive account of equity, or distributive justice, in health (including the contributions of ethicists, philosophers and public health professionals) is available [31].

It is important to realise that the measurements of inequality above are provided in the absence of data about mental health status, or of any other factors that may be relevant to the level of inequality. In other words, only univariate measures of inequality are provided here. The multivariate factors that explain psychiatric services utilisation rates are not considered in these measures. Thus, in the absence of such data, it is not possible to draw strong inferences about the nature of the equality reported above.

Inferences about the fairness of the distributions are inappropriate. For example, the distribution PS_t = 3_, while appearing to be more equal, may not be fairer, in the absence of data about medical diagnoses for the individuals in that population. PS_t = 1_, PS_t = 2 _and PS_t = 4 _may actually be more equal and fairer distributions of services, if the increase in service use is associated with increments of reducing levels of mental health. However, if distributions, PS_t = 1_, PS_t = 2_, PS_t = 3 _and PS_t = 4_, refer to populations of uniform mental health status, then these Lorenz curve enrich our insight into the nature of the inequality.

Which of the above measures is the "correct" measure? That question is answered by emphasising again that value judgments exist with all measures. Consider, for example, the coefficient of variation. This measure values all reductions in inequality equally. That is, in the context of income, a transfer between time period 1 and time period 2 from a millionaire to a semi-millionaire is equally weighted as a transfer of the same amount from a millionaire to the poorest person during the two time periods. All indexes of inequality interpret the magnitude of equality in such a transfer differently, and there are limitations with each measure. Recall that it is not possible to interpret intersecting Lorenz curves. Furthermore, the value of a Gini co-efficient is more sensitive to changes in the middle of the income distribution than to changes in the lower or higher tails of the same distribution. More detailed discussions of the properties of inequality measures are available [7,18,20].

Thus, it is desirable in inequality measurement to employ several measures, subject to the constraints of data. Doing so also makes inequality rankings explicit. Summary statistics can produce conflicting inequality rankings, a point that has been shown repeatedly in the income distribution literature. See Atkinson's elegant illustration concerning the income distribution of seven advanced and five developing countries [16]. He provides 66 pair-wise comparisons, not only with summary statistics, but also with Lorenz curves, Gini coefficients and coefficients of variation, and finally Atkinson measures with three assumptions of ε. He reveals differing rankings in terms of inequality from those measures.

The point of these discussions is this: it is often important in measuring inequality to report several measures/indexes, within the constraints of the data available, and to examine the strengths and weaknesses of each measure. In so doing, the nature of the inequality is depicted more accurately, and one can weigh equity judgements more wisely, than is possible by emphasising any single measure of inequality.

## Conclusion

Equality and equity are not interchangeable terms. Conceptually, they are dissimilar and the techniques that provide evidence about each of these contrasting aspects of distributions are not the same. Discussion of various distributions of health sector variables has resounded "loud and long", both at the time of the formation of Welfare State policies after World War II, and again towards the end of the twentieth century. However, in Australia the body of empirical evidence about distribution in the health sector is minute, the terms "equality" and "equity" are used interchangeably at times, and the issues are often discussed in the absence of empirical evidence.

This paper has been concerned with how to provide useful empirical evidence about one distribution in the health sector, that of psychiatric services, although applications throughout the entire health sector are possible. The paper has demonstrated the usefulness of standard economic concepts and techniques in distribution measurement. Measurement of inequality involves measuring both the location of a distribution, such as the mean, the median and so forth, and measuring the dispersion of the distribution in terms of "univariate" measures, such as the standard deviation, the coefficient of variation, the Gini coefficient, the Lorenz curve, along with welfare-based measures, such as Atkinson's measure. So-called bivariate measures do not measure the distribution *per se*.

It is important to apply several measures of equality to a data set, when measuring distributions. Applying only a single measure of inequality may produce misleading results because different value judgements underlie every measure. Economic concepts are attached to all statistical measures and, when comparing distributions at differing places or through time, a "raft" of measures should be applied in order to measure and present the distributions fully from various angles and value judgements.

Measuring the equality of a distribution of psychiatric services is an exercise in descriptive economics. It is thus just one aspect of examining distributional issues. This paper used some simple data to illustrate the measurement of distributions of psychiatric services. Some of these distributions were determined to be relatively more equal. Of course, relatively equal distributions of psychiatric services are not necessarily equitable, nor are relatively unequal distributions equitable. Evidence about mental health status, age, gender, location, income or such information as is relevant in judging equity, would be required for such conclusions.
